# 

**Published:** 1882-12

**Authors:** 


### To The Ohio State Dental Society.

Arrangements have recently been made with the various railroads centering at Columbus to sell excursion tickets to those attending the Society, at reduced rates. Tickets for the round trip are to be obtained at the point of starting upon a certificate, which can be secured by addressing the President, Dr. A. F. Emminger, of Columbus, O., or the Secretary, Dr. W. H. Sillito, Xenia, O. These must be secured before starting from home. Bear in mind the meeting is on Wednesday, Dec. 6th at 10 A.M.

### INDEX TO VOLUME XXXVII.

A New Dental disease.............. 46

A New Dental College ............ 47

A General Treatment of Irregularities ......................... 24

A Singular Suicidal Attempt ... 83

Apthous Sore Mouth of Infants 85

A Good Bracket ...................... 100

A New Journal......................... 100

Anæsthetics Medico — Legally Considered.......................... 103

Ammonia—Its Relations to Dentistry................................ 133

A Case in Practice.................. 142

Address to Graduating Class..... 179

Another Resolution ............... 203

An Important Step ................ 256

American Dental Association... 258

Aristocracy of Professions........ 297

Address ................................. 300

American Dental Association... 307

A Manual of Dental Anatomy.. 308

Arrangements for the Meeting of the American Dental Association ..................... 314

American Dental Association... 351

Action of Chloroform.............. 355

American Medical Association.. 359

American Dental Association ..................................364–423

Alveolar Abscess..................... 383

Aristocracy of Profession ...389–420

Alimentation........ ................... 392

Annual Address...................... 402

A Suggestion for the Laboratory 461

A New Phase of the Amalgam Question.......................... 500

Application of Peroxide of Hydrogen for Medical Purposes ............................. 508

Absence ......... ...... .............. 522

Biographical—Dr. M. S. Dean .. 145

" Dr. D. C. Hamphurst.. 153

Chemistry in the Practice of Dentistry ........................ 261

Changes .................................. 312

Causes of Complaint Against Vaccination..................... 357

Correlated Diseases of the Teeth and Ear............................. 365

Cautery .................................. 460

Crural Neuralgia Among Dentists .................................. 565

Counter Irritation.................. 566

Continuous Gum Dentures........ 569

Correspondence..................615–617

Development of the Enamel..... 62

Development of the Eyes......... 83

Definite Causes of Caries......... 175

Dental Societies... ................. 201

Dental Pathology and Surgery.. 226

Dental Diploma in the State of New York ...................353–407

Difficulties Arising from Eruption of a Third Molar ...... 409

Death of Dr. E. M. Nutting..... 411

Dental Tumor of Jaw.............. 458

Died—Miss Belle Palmer......... 522

Extraction of Teeth in Pregnant Women ............................ 205

Editorial ................................ 619

Exposed Pulp—Treatment and Filling ......... ................. 323

Female Students at the Pennsylvania Hospital .................. 81

Fistula Beneath the Chin......... 148

From F. S. C. Edwards, Mississippi ................................ 305

Functional Habitude.............. 315

General Acceptance of Antiseptic Methods........................ 85

Gone Hence.......................... 102

Going Abroad......................... 364

Hemorrhage and Gangrene from Carious Tooth.................. 457

How Printing Affects the Health ............................ 88

Home Again.......................... 204

Hypodermic Administration of Carbolic Acid .................. 246

How to Apply the Soda Remedy in Burns and Scalds........... 459

Illinois Dental Society.........202–259

Improvement in Plate Teeth..... 619

In Memoriam......................... 151

Indiana State Dental Society ....................................310–412

I Will Go a Fishing ............... 415

Impure Water Makes Impure Ice.................................. 511

Kansas State Dental Association 202

Kentucky State Dental Association ..............................311-448

Letter from Dr. E. G. Betty ..... 355

Light—Vision ....................... 518

Mortality Statistics ................. 50

Michigan Dental Association ... 152

Manual of Dental Surgery and Pathology......................... 313

Manual of Dental Surgery and Pathology ........................ 313

Methods and Manipulations with Gold in Filling Teeth 376

Mysticism ............................. 611

Nature's Royal Family............ 207

National Dental Association..... 351

New Method of Preparing the Spinal Cord for Microscopic Sections ........................... 461

On Specialism........................ 1

On the Remedies Used to Promote Absorption of Inflammatory and Other Morbid Products.......................... 9

Our Medical Literature............ 13

On the Cause of Death Under Chloroform ....................... 86

Obituary ............................... 101

Operative Dentistry................ 242

Osseous Structure of the Teeth.. 268

On the Causes of Dental Caries.. 417

Otalgia from Reflex Irritation.. 461

Obituary ............................... 468

On the Filling and Treatment of the Permanent Molar Teeth Between the Ages of Six and Fourteen .................. 480

On the Process of Absorption in Bone and Tooth Structure.. 484

On Nicotine and its Action Upon the Teeth......................... 496

Ohio State Dental Society ........ 570

Proceedings of the Michigan State Dental Society ......... 330

Proceeding of the Eighteenth Annual Meeting of the Illinois State Dental Society 339

Petition to the Municipal Council of Paris, France........... 361

Pennsylvania State Dental Society ........................... 364

Physicians' Visiting List......... 622

Poulticing ............................. 465

Peroxide of Hydrogen in Surgery.................................. 568

Queries................................. 410

Relative Mortality Amongst White and Colored Races .. 46

Risorcine and its Employment in Therapeutics ............... 309

Reflex Lesions of the Mouth, Associated with Pregnancy 469

Salycilic Acid as a prophylactic 44

Sanitary ................................ 49

Sanitary Condition and Longevity of Jews ................ 81

Sir Erasmus Wilson ............... 88

Some Hints on the Preparation and Mounting of Microscopic Objects ..............123–289

Soldering in Dentistry............ 607

Sanguinary Calculus............... 466

Sulphurous Acid as a Disinfectant.................................. 510

Success in the Profession of Dentistry ......................... 554

Students' Manual of Histology.. 621

Sir Erasmus Wilson's Munificent Gift to Margate......... 569

The Medical and Dental Laws.. 49

The Innervation of the Salivary Glands .......................... 43

Transmission of Traumatic Lesion.......................... 45

To Prevent Plaster from Adhering to Rubber Plates in Vulcanizing .................... 87

The Effects of Ozone .............. 89

The General and Special Education of Dentists ............ 90

The Dentist of Bigler Gulch..... 77

The Ohio State Journal of Dental Science ...................... I01

The Annual Meeting of the Oldest Dental Society............. 99

The Value of Mental Tension... 80

The Freezing Cure.................. 150

Transactions ........................... 151

The Essential Oils .................. 165

The Dental Pulp—Its Pathology and Treatment................ 155

The Graduates....................... 204

The Relation of Dentistry to Medicine ........................ 219

The Disorders of Primary Dentition.............................. 231

The Cause and Prevention of Dental Caries.................. 237

The Management of Deciduous Teeth ............................. 276

The Relative Value of the Cuspidati ........................ 282

To the Profession.................... 345

The American Dental Association .............................. 359

The Tail of the Beast............. 362

The Freezing Cure.................. 411

Transaction of American Association .......................... 416

Treatment of the Deciduous Teeth ............................ 601

The Administration of Chloroform ................................ 463

To be Resumed — The Mad River Dental Society......... 467

The Cause of Education........... 506

The Colleges.......................... 520

The Centralization of Energy... 523

The Etiology and Pathology of Dental Diseases ............... 546

Teeth ................................... 516

The Topical Uses of Tannic Acid ............................... 567

Transactions—Michigan State Dental Society.................. 572

Transactions—Ohio State Dental Society...................... 572

Writer's Cramp..................... 12

Wheat Phosphates.................. 30

Wooden Toothpicks ............... 306

Wisconsin Denial College Again 360

Why Not............................. 414

Vulcanizers and Vulcanizing ... 303

### DIRECTORY OF DENTAL SOCIETIES.

*American Dental Association*—Meets on the 1st Tuesday of August 1882, in Cincinnati, Pres. H. A. Smith. Cincinnati, O.; Cor. Sec. M H. Webb, Lancaster, Pa.; Rec. Sec. Geo. H. Cushing, Chicago, Ill.

*National Dental Association*—Meets Thursday, in Washington City, Aug. 3, 1882. Pres. Jno. B. Rich, New York; Sec. R. Finley Hunt, Washington, D. C.

### STATE DENTAL SOCIETIES.

*Alabama Dental Association*—Meets annually. Next meeting at Montgomery, on the second Tuesday of April, 1882. Pres. G. M. Rousseau, Montgomery; Sec., T. M. Allen, Eufula.

*California State Dental Association*—Tuesday, June 1st, 1882. Pres. S. E. Bowles, San Francisco; Sec. S. E. Goe., San Francisco; Cor. Sec. H. E. Knox, San Francisco.

*Connecticut State Dental Society*—Pres. E. T. Payne; Rec. Sec. Geo. L. Parmelee; Hartford. Semi-annual meeting in October, 1882, at Hartford.

*Dental Society of the State of Maryland and District of Columbia*—Meets annually. Next meeting at Washington City, on the second Tuesday in October, 1882. Pres. B. F. Coy, of Baltimore: Sec. M. W. Foster. M. D

*Georgia State Dental Society*—Meets second Tuesday in May, 1882, at Macon. Pres. W. W. Ford, Macon, Rec. Sec. R. A. Holliday, Atlanta, Cor. Sec. L. D. Carpenter, Atlanta.

*Illinois State Dental Society*—meets annually. Pres. A. W. Harlan, Chicago; Sec. E. Noyes, Chicago. Next place of meeting, Quincy, second Tuesday in May, 1882

*Indiana State Dental Society*—Meets next year at Indianapolis, on the last Tuesday of June, 1882. Pres. Robert Van Valzah, Terre Haute; Sec. W. N. Hall, Terre Haute.

*Iowa State Dental Society*—Meets annually. Next meeting at Des Moines, on the first Tuesday in May, 1882. Pres. G. W. Fuller, Des Moines; Rec. Sec. E. E. Hughes, of Newton; Cor. Sec. A. O. Hunt, McGregor.

*Kentucky State Dental Association*—Meets annually, first Tuesday in June, 1882. Next meeting in Lexington. Pres. C. G. Edwards, Louisville; Sec. W. W. Justice, Winchester.

*Kansas State Dental Association*—Pres. A. Doud, Sec. J. A Young, of Leavenworth: Next meeting will be held at Topeka, first Tuesday in May, 1882.

*Maine Dental Society*—Meets in August, at Thomaston. Pres. E. J. Robert, Agusta, Sec. C. E Hussey, Beddeford.

*Massachusetts Dental Society*—Meets semi-annually. Pres. G. F. Waters, Boston, Rec. and Cor. Sec. W. E. Page, Charlestown.

*Michigan State Dental Association*—Meets annually. Next meeting at Detroit, last Thursday in March, 1882. Pres. A. T. Metcalf, Kalamazoo: Sec. M. F. Finley, Ypsilanti: Treas. J. Lathrop, Detroit.

*Mississippi State Dental Association*—Will meet third Tuesday of January, 1882, at Jackson. Pres. J. D. Miles, Vicksburg; Sec. G. W. Rembert, Natchez.

*Minnesota Dental Association*—Meets annually. Meets May 26, 1882, at Winona. Pres. W. F. Lewis, Sec C. M. Bailey, Minneapolis.

*Missouri Dental Association*—Meets annually on the first Tuesday of June, 1882. Next meeting at Sweet Springs. Pres. A. N. Fuller; Sec. W. H. Eames, St. Louis.

*Nebraska State Dental Society*—Meets annually. Next meeting July 23, 1882, at Fremont. Pres. J. W. Chadduck, Nebraska City; Sec. W. F. Roseman, Fremont.

*New Jersey State Dental Society*—Meets annually. Next meeting at Long Branch. on Wednesday, July, 20th 1882. Pres. F. A. Levey, Orange; Sec. C. A. Meeker, Newark.

*New Hampshire Dental Society*—Pres. D. W. Eagerly, Farmington; Sec. C. W. Clerment, Manchester.

*North Carolina State Dental Society*—Meets annually. Next meeting at Raleigh, first Wednesday in September, 1882. Pres. V. E. Turner; Sec. D. A. Robinson.

*Ohio State Dental Society*—Meets annually at Columbus, first Wednesday of December, 1882. Pres. A. E. Emminger, Columbus; Rec. Sec. W. H. Sillito, Xenia.

*Oregon State Dental Society*—Meets annually. Pres. J. R. Cardwell, Portland; Rec. Sec. S. J. Barber.

*Pennsylvania State Dental Society*—Meets Tuesday, July 27th, 1882, at Chatauqua Lake; Pres. H. Gerhart, Lewisburg; Rec. Sec. E. P. Kremer, Lebanon, Pa.; Cor. Sec. E. B. Long, Pittston.

*Rhode Island Dental Association*—Pres. A. W. Buckland, Woonsocket; Sec. L. L. Buckland, Providence.

*South Carolina State Dental Association*—Meets annually. Pres. B. A. Muckenfuss; Sec. G. F. S. Wright, Columbia; Cor. Sec. R. A. Smith. Next meeting at Cheraw, May 3, 1882.

*Texas State Dental Association*—Meets in the city of Austin, May 4, 1882. Pres.——— Sec. W. R. Clifton

*Tennessee Dental Association*—Meets Semi-annually. Next meeting at Nashville, first Tuesday in February, 1882. Pres. A. F. Claywell, Lebanon; Rec. Sec. H. W. Morgan, Nashville; Cor. Sec. R. R. Freeman, Nashville.

*The Dental Society of the State of New York*—Meets annually on the second Wednesday in May. Next session at Albany, May 12, 1882. Pres. C. E. Francis, New York; Rec. Sec. S. A. Freeman, Buffalo; Cor. Sec. W. H. Atkinson, New York.

*Vermont State Dental Society*—Meets annually. Next meeting at Burlington, March 15, 1882. Pres. L. T. Lawton, Rutland, Sec. C. F. Lewis, Burlington.

*Virginia State Dental Association*—Meets in Charlottsville, Tuesday, August 19, 1882. Pres. J. Hall Moore; Sec. L. M. Cowarden, Richmond.

*Wisconsin State Dental Association*—Meets annually. Next meeting at Milwaukee, July 20, 1882. Pres. M. T. Moore, La Crosse; Sec. Geo. H. McCansey, Janesville.

### LOCAL SOCIETIES.

*American Academy of Dental Science*—Meets annually in Boston, Mass., on the last Wednesday in October, 1881. Pres. J. L. Williams; Rec. Sec. Jno. T. Codman; Cor. Sec. C. P. Wilson, Boston.

*Brooklyn Dental Society*—Meets monthly. Pres. Chas. D. Cooke; Cor. Sec. W. H. Atkinson. Rec. Sec. A. P. Crandall, 508 Clinton Ave., Brooklyn.

*Central Dental Association of Northern New Jersey*—Meets annually in February. Pres. C. A. Meeker, Newark, Sec. G. Carleton Brown, Elizabeth.

*Cincinnati Dental Society*—Meets on the second Tuesday in each month. Pres. W. S. How; Sec. A. G. Rose.

*Connecticut Valley Dental So iety*—Meets 27 and 28 of Oct. 1882, at Savin Rock Conn. Pres. C. T. Stockwell, Springfield, Mass. Sec. A. M. Ross, Chicopee.

*East Tennessee Dental Association*—Meets annually. Next meeting will be held at Chattanooga, Tenn., on the first Tuesday of Aug., 1882. Pres. W. F. Fowler, Greenville; Sec. W. M. Snyder, Athens.

*New Hampshire State Dental Society*—Meets annually. Pres. H. Hill, Manchester; Sec. E. B. Davis, Concord.

*Merrimack Valley Dental Association*—Meets annually. Pres. J. N. Chandler, Boston; Sec. H. W. Coburn, Lowell, Mass.

*Mississippi Valley Dental Society*—Meets annually at Cincinnati, on first Wednesday in March, 1882. Pres. J. W. Jay, Richmond; Sec. N. S. Hoff, Cincinnati.

*Nashville Dental Association*—Meets on the second Tuesday of each month, Pres. R. R. Freeman; Sec. L. G. Noel, Nashville.

*Northern Ohio Dental Association*—Meets annually. Next meeting at Cleveland. on the second Tuesday in May, 1882. Pres. C. Buffett, Cleveland, O.; Rec. Sec. A. J Douds, Canton: Cor. Sec. H. F. Harvy, Cleveland, O.

*Ohio Dental College Association*—Meets annually, Tuesday, March 2, 1882, at Cincinnati. Pres. H. M. Reid, Cincinnati; Rec. Sec. F. A. Hunter, Cincinnati.

*Odontographic Society of Pennsylvania*—Meets on the first Wednesday of each month, at 8 o'clock p. m., in the Philadelphia Dental College. Pres. J. L. Eisenbrey, Rec. Sec. E. L. Hewitt.

*Odontological Society of New York City*—Meets the second Tuesday of each month. Pres. W. A. Bronson; Rec. Sec. Wm. Jarvie, Jr.

*Pennsylvania Association of Dental Surgeons.*—Pres. J. H. Githens. Rec. Sec. Theo. F. Chupein.

*Pittsburg Dental Society*—Meets the second Tuesday Evening of each month. Pres. N. W. Aithen; Sec. L. Depriz.

*Fifth District Dental Society of N. Y.*—Meets semi-annually. Next meeting in Syracuse, on the 12th of October, next. Pres. E. L. Swartwout, of Utica. Sec. I. C. Curtis of Fulton.

*San Francisco Dental Society*—Meets monthly. Pres. Alex. Warner: Sec. A. W. Knowles; Treas. J. J. Birge.

*Sixth District Dental Society of New York*—Meets semi-annually. Next meeting at Cortland, Thursday, Oct. 6th, 1882. Pres. L. E. Freeland, Onconta; Sec. A. J. Wright, Oswego.

### EXCHANGES.

St. Louis, Medical and Surgical Journal.
The Pacific Medical and Surgical Journal, San Francisco.
Cincinnati Medical Advance.
Cincinnati Lancet and Clinic.
Dental Cosmos, Philadelphia.
Chicago Medical Examiner.
The Detroit Review of Medicine and Pharmacy.
Medical Record, N. Y.
American Journal of Dental Science, Baltimore.
Indiana Journal of Medicine.
Missouri Dental Journal, St. Louis.
The Sanitaran, New York City.
The Monthly Review of Dental Surgery, London.
L'art Dentaire, Paris.
Le Progress Dentaire, Paris.
Deutische Verteljahrsschrift, Leipzig.
Physician and Surgeon, Ann Arbor, Mich.
Ohio State Journal of Dental Science, Toledo, Ohio.
" " " " " " Xenia, O.
The Microscope, Ann Arbor, Mich.
Scientific American, New York.
Independent Practitioner, N. Y.
Dental Miscellany, N. Y.
The Dental Record, London.

### UNIVERSITY OF CALIFORNIA—DENTAL DEPARTMENT.

### FACULTY.

W. T. REID, A.M., President of the University, and Ex-Officio President of the Faculty.
A. F. McLAIN, M.D., D.D.S., Professor of Dental Pathology and Therapeutics.
S. W. DENNIS, M.D., D.D.S., F.R.M.S., Professor of the Principles and Practice of Operative Dentistry and Dental Histology.
C. L. GODDARD, A.M., D.D.S., Professor of Mechanical Dentistry.
M. W. FISH, M.D., Professor of Physiology.
A. W. PERRY, M.D., Professor of Chemistry.
WILLIAM LEWITT, M.D., Professor of Anatomy.
W. E. TAYLOR, M.D., Professor of the Principles and Practice of Surgery.

### DEMONSTRATORS.

..........................., Demonstrator of Operative Dentistry.
..........................., Demonstrator of Mechanical Dentistry.
W. B. LEWITT, M.D., Demonstrator of Anatomy.
E. O. COCHRAN, Demonstrator of Continuous Gum Work.
M. J. SULLIVAN, D.D.S., Demonstrator of Salivary Analysis.

### CLINICAL INSTRUCTORS.

J. A. W. LUNDBORG.

H. E. KNOX, D.D.S.

R. E. COLE, D.D.S

R. CUTLAR, D.D.S.

GEO. OTIS LAWRENCE, D.M.D.

H. J. PLOMTEAUX.

WM. B. KINGSBURY.

H. C. DAVIS, L.D.S.

R. W. HENDERSON.

J. L. ASAY, M.D.

J. H. HATCH, D.D.S.

L. L. DUNBAR, D.D.S.

JOHN RABE, D.D.S.

Surgical and Medical Clinics are held at the Hospitals three times a week.

The Lecture Rooms, Operating Rooms, and Laboratories are commodious, and their appointments complete.

The Preliminary Course of Lectures will commence April 1st, 1882. The Regular Course will commence June 1st, 1882, and continue till October 30th.

Students may matriculate at any time, but a preliminary examination will be required of all matriculates after the expiration of the first year.

Two years' study, attendance upon two courses of lectures, and examination at the end of the second course are required for graduation.

Students who have attended one course of lectures at any other Dental School in good standing, and Dentists of seven years' practice may be examined for the degree of D.D.S. after attending a single course of lectures.

Graduates of medicine may apply for the degree of D.D.S. upon attending one full year.

### FEES.

| | | | |
|---|---|---|---|
| Matriculation Fee, - - | $5.00. | Demonstrators' Fees, - - | $30.00. |
| Tuition, - - - - | $100.00. | Diploma Fee, - - - | $30.00. |

For further information, address

***S. W. DENNIS, Dean, No. 33 Kearney Street, San Francisco, Cal.***

N. B.—BOARDING.—The expense of living in the city of San Francisco is not great. Good board, with room rent, may now be procured at the low rate of $5.00 per week, at a convenient distance from the College building. Students from a distance may learn the address of these boarding houses, and other information, by calling on the Dean of the Faculty.

Jan. 82-1y.

### UNIVERSITY OF CALIFORNIA—DENTAL DEPARTMENT.

### FACULTY.

W. T. REID, A.M., President of the University, and Ex-Officio President of the Faculty.

A. F. McLAIN, M.D., D.D.S., Professor of Dental Pathology and Therapeutics.

S. W. DENNIS, M.D., D.D.S., F.R.M.S., Professor of the Principles and Practice of Operative Dentistry and Dental Histology.

C. L. GODDARD, A.M., D.D.S., Professor of Mechanical Dentistry.

M. W. FISH, M.D., Professor of Physiology.

A. W. PERRY, M.D., Professor of Chemistry.

WILLIAM LEWITT, M.D., Professor of Anatomy.

W. E. TAYLOR, M.D., Professor of the Principles and Practice of Surgery.

### DEMONSTRATORS.

..........................., Demonstrator of Operative Dentistry.

..........................., Demonstrator of Mechanical Dentistry.

W. B. LEWITT, M.D., Demonstrator of Anatomy.

E. O. COCHRAN, Demonstrator of Continuous Gum Work.

M. J. SULLIVAN, D.D.S., Demonstrator of Salivary Analysis.

### CLINICAL INSTRUCTORS.

J. A. W. LUNDBORG.

H. E. KNOX, D.D.S.

R. E. COLE, D.D.S

R. CUTLAR, D.D.S.

GEO. OTIS LAWRENCE, D.M.D.

H. J. PLOMTEAUX.

WM. B. KINGSBURY.

H. C. DAVIS, L.D.S.

R. W. HENDERSON.

J. L. ASAY, M.D.

J. H. HATCH, D.D.S.

L. L. DUNBAR, D.D.S.

JOHN RABE, D.D.S.

Surgical and Medical Clinics are held at the Hospitals three times a week.

The Lecture Rooms, Operating Rooms, and Laboratories are commodious, and their appointments complete.

The Preliminary Course of Lectures will commence April 1st, 1882. The Regular Course will commence June 1st, 1882, and continue till October 30th.

Students may matriculate at any time, but a preliminary examination will be required of all matriculates after the expiration of the first year.

Two years' study, attendance upon two courses of lectures, and examination at the end of the second course are required for graduation.

Students who have attended one course of lectures at any other Dental School in good standing, and Dentists of seven years' practice may be examined for the degree of D.D.S. after attending a single course of lectures.

Graduates of medicine may apply for the degree of D.D.S. upon attending one full year.

### FEES.

| | | | |
|---|---|---|---|
| Matriculation Fee, - - | $5.00. | Demonstrators' Fees, - - | $30.00. |
| Tuition, - - - - | $100.00. | Diploma Fee, - - - | $30.00. |

For further information, address

*S. W. DENNIS, Dean, No. 33 Kearney Street, San Francisco, Cal.*

N. B.—BOARDING.—The expense of living in the city of San Francisco is not great. Good board, with room rent, may now be procured at the low rate of $5.(0 per week, at a convenient distance from the College building. Students from a distance may learn the address of these boarding houses, and other information, by calling on the Dean of the Faculty. Jan. 82–1y.

### THE DENTAL COLLEGE

OF THE

### University of Michigan.

The Eighth Annual Session of this Institution will commence on the 1st of October and close cn the last Wednesday of March, thus making a course of six months. The regular course of instructions commences at once, and will proceed through the term, with the customary vacation.

The students in this department will receive instructions in Anatomy, Physiology, Pathology, Chemistry, Materia Medica, Therapeutics, and Surgery, from the Professors of their respective branches in the *Department of Medicine and Surgery* of the University, when lectures commence, and continue the same as with the Dental College. There will also, in addition, be a special course upon each of these branches. The courses will each embrace from ten to fifteen lectures.

### FACULTY OF THE DENTAL DEPARTMENT.

| | |
|---|---|
| J. B. ANGELL, LL.D. | President. |
| J. TAFT, M.D., D.D.S. | Principles and Practice of Operative Dentistry. |
| CORYDON L. FORD, M.D., D.D.S. | Anatomy and Physiology. |
| J. A. WATLING, D.D.S. | Clinical and Mechanical Dentistry. |
| W. H. DORRANCE, D.D.S. | Prosthetic Dentistry. |
| U. D. BILLMEYER, D.D.S. | Demonstrator of Clinical Dentistry. |
| C. S. CASE, D.D.S. | Demonstrator of Prosthetic Dentistry. |

Special instruction will be given in Dental Pathology, Oral Surgery, Dental Therapeutics, and Diseases of Women and Children, with reference to the teeth.

Students should be promptly present at the College on Friday, September 29th, at 10 o'clock, A.M., to make the preliminary arrangements for entering upon regular work. Seats in the lecture room are assigned by selection to students in the order of registration on the Steward's Books; and each student is expected to occupy during the session such seat as he may select. Students, on arriving in Ann Arbor, should call at the Steward's office.

### CONDITIONS OF GRADUATION.

The candidate must be twenty-one years of age. *He must furnish satisfactory evidence of good moral character.*

He must devote three years to the study of his profession. He must attend two full courses of lectures in the Dental College, or one course in some college having an equal standard of requirements, and the last one here, and we recommend that he attend three courses regularly.

He must sustain an examination satisfactory to the Faculty in all the branches taught.

A graduate of the Medical College may enter the Senior Class, and, if found qualified, may graduate after one year has been devoted to the study of dentistry.

### FEES AND EXPENSES.

The fees, which must be paid in advance, are as follows:

RESIDENTS OF MICHIGAN.—Matriculation fee, $10.00; annual dues, $20.00

NON-RESIDENTS.—Matriculation. $25.C0; annual dues, $25 00.

GRADUATION FEE.—For all alike, $10.00. The admission fee is paid but once, and entitles the student to the privileges of permanent membership in any department of the University. The annual due is paid the first year and every year thereafter while at the University.

For further particulars, address the Dean of the Dental College, Ann Arbor, Michigan.

**J. TAFT, Dean.**

May-82-6m.

### THE DENTAL COLLEGE

OF THE

### University of Michigan.

The Eighth Annual Session of this Institution will commence on the 1st of October, and close on the last Wednesday of March, thus making a course of six months. The regular course of instruction commences at once, and will proceed through the term, with the customary vacation.

The students in this department will receive instructions in Anatomy, Physiology, Pathology, Chemistry, Materia Medica, Therapeutics, and Surgery, from the Professors of their respective branches in the *Department of Medicine and Surgery* of the University, when lectures commence, and continue the same as with the Dental College. There will also, in addition, be a special course upon each of these branches. The courses will each embrace from ten to fifteen lectures.

### FACULTY OF THE DENTAL DEPARTMENT.

| | |
|---|---|
| J. B. ANGELL, LL.D. | President |
| J. TAFT, M.D., D.D.S. | Principles and Practice of Operative Dentistry |
| CORYDON L. FORD, M.D., D.D.S. | Anatomy and Physiology |
| J. A. WATLING, D.D.S. | Clinical and Mechanical Dentistry |
| W. H. DORRANCE' D.D.S. | Prosthetic Dentistry |
| U. D. BILLMEYER, D.D.S | Demonstrator of Clinical Dentistry |
| C. S. CASE, D.D.S. | Demonstrator of Prosthetic Dentistry |

Special instruction will be given in Dental Pathology, Oral Surgery, Dental Therapeutics, and Diseases of Women and Children, with reference to the teeth.

Students should be promptly present at the College on Friday, September 29th, at 10 o'clock, A.M., to make the preliminary arrangements for entering upon regular work. Seats in the lecture room are assigned by selection to students in the order of registration on the Steward's Books; and each student is expected to occupy during the session such seat as he may select. Students, on arriving in Ann Arbor, should call at the Steward's office.

### CONDITIONS OF GRADUATION.

The candidate must be twenty-one years of age. *He must furnish evidence of good moral character.*

He must devote three years to the study of his profession. He must attend two full courses of lectures in the Dental College, or one course in some college having an equal standard of requirements, and the last one here, and we recommend that he attend three courses regularly.

He must sustain an examination satisfactory to the Faculty in all the branches taught.

A graduate of the Medical College may enter the Senior Class, and, if found qualified, may graduate after one year has been devoted to the study of dentistry.

### FEES AND EXPENSES.

The fees, which must be paid in advance, are as follows:

Residents of Michigan.—Matriculation fee, $10.00; annual dues, $20.00.

Non-Residents.—Matriculation, $25.00; annual dues, $25.00.

Graduation Fee.—For all alike, $10.00. The admission fee is paid but once, and entitles the student to the privileges of permanent membership in any department of the University. The annual due is paid the first yenr and every year thereafter while at the University.

For further particulars, address the Dean of the Dental College, Ann Arbor, Mich.

**J. TAFT, Dean.**

May-82-6m

### CONTENTS.

### COMMUNICATIONS.

A MONTHLY MAGAZINE DEVOTED TO THE INTERESTS OF THE

### DENTAL PROFESSION.

*Terms Reduced to $2.00 Per Year. Single Copies, 25 Cents.*

### EDITED BY PROF. J. TAFT, D.D.S.

—AND—

**Published by SPENCER & CROCKER, Ohio Dental Depot.**

The volume commences in January and closes in December.

The REGISTER being issued monthly is always fresh, therefore Indispensable to the practitioner who desires to keep posted up with the new inventions and improvements which are daily developed. Neither expense nor effort will be spared to give value and variety to its contents. Articles will be illustrated with well executed engravings whenever they will add to the interest or assist to explanation.

Its extensive circulation and frequency of issue make it one of the BEST DENTAL ADVERTISING MEDIUMS in the United States.

All subscriptions must begin with January or July.

Local or special notices, five lines or less, will be inserted for $3 each insertion; over five lines, 50 cts. per line.

All advertising bills payable in advance, or at furthest, after the issue of the first number containing the advertisement. All transient advertisements to be paid for in advance.

☞CONTRIBUTIONS SOLICITED.

Exclusively Editorial Communications should be addressed to the Editor.

The latest number of the REGISTER may be ordered with or without other goods at any time, and all letters should be addressed to

**SPENCER & CROCKER, Ohio Dental Depot, Cincinnati, O.**

